# Sexual conflict and sexual networks in bed bugs: the fitness cost of traumatic insemination, female avoidance and male mate choice

**DOI:** 10.1098/rspb.2023.2808

**Published:** 2024-07-17

**Authors:** Janice L. Yan, Maggie L. Dobbin, Reuven Dukas

**Affiliations:** ^1^ Animal Behaviour Group, Department of Psychology, Neuroscience & Behaviour, McMaster University, Hamilton, Ontario, Canada

**Keywords:** bed bugs, male mate choice, polyandry, sexual conflict, traumatic insemination, social network analysis

## Abstract

Sexual conflict is prevalent among animals and is primarily caused by the fact that the optimal mating rates are often higher in males than in females. While there is a growing appreciation that females can also gain from multiple matings, we still know relatively little about which sex controls the observed mating rates and how close it is to the optimal female mating rates. To address this issue, we tracked female bed bugs (*Cimex lectularius*) inseminated daily versus weekly and found that weekly inseminated females lived longer and produced over 50% more offspring. In a follow-up experiment employing a social network framework, we placed 24 bed bugs into a semi-naturalistic arena and recorded all sexual interactions. While recently inseminated females did not avoid males more often, they were more frequently rejected by males. Finally, we tracked avoidance behaviour in a single cohort of female bed bugs as they received six successive daily inseminations. Avoidance rates increased and insemination durations decreased with increasing number of prior inseminations. Overall, our results indicate high costs of polyandry. Although females possess some plastic avoidance strategies, the observed rates of insemination fall closer to the male rather than female optimum.

## 1. Introduction

Sexual conflict occurs when the reproductive interests of males and females are at odds with one another [1,2]. This conflict is prevalent among animals and manifested in sex-specific traits that provide benefits to one sex at the detriment of the other [2–4]. A common cause for sexual conflict is the fact that the optimal mating rate is typically higher in males than in females, which leads to males often pursuing and even coercing reluctant females into mating [5–7]. Examples of sexually antagonistic traits that benefit males at the expense of females include the elaborate morphological structures of male water striders (*Gerris odontogaster*) that have evolved for grasping resistant females [8,9] and seminal fluid proteins, which enhance male paternity share but decrease female survival [10–13] . While sexual conflict is well documented in many species, there is now a growing appreciation that the optimal mating rates of females are higher than previously thought. Even though females may be able to produce offspring for the rest of their lives after a single mating, some intermediate mating rates may balance the costs and benefits to females, and hence maximize their lifetime reproductive success [7,14–16].

Although females may gain from multiple matings, we still know relatively little about their optimal mating rates and how they compare to naturally observed mating rates. If observed mating rates are determined by males, they may exceed the rates that are optimal for females. To address this issue, we conducted a series of experiments using bed bugs (*Cimex lectularius*) as a model system. Bed bug reproduction involves obligatory traumatic insemination, whereby males bypass females’ genital tracts and instead use their needle-like intromittent organs to pierce female abdomens and insert sperm [17–20]. Although traumatic insemination has independently evolved multiple times and is prevalent among a wide variety of animals, its evolutionary biology is not well understood [20,21]. Traumatic insemination in bed bugs involves a fitness cost to females owing to the injury and subsequent immune response and healing [19,22,23]. Despite such costs and the fact that female bed bugs remain fertile for about 9.5 weeks after a single insemination [24], the average insemination rate of female bed bugs in semi-natural settings is approximately once per day [25]. This suggests sexual conflict over insemination rates, which we assessed in three experiments designed to quantify the fitness consequences of low- and high-insemination rates, and the behaviours of each sex that lead to the high insemination rates observed under semi-natural settings.

First, we compared the longevity, egg production rates, egg viability and offspring production rates of females under low and high traumatic insemination rates informed by our data from semi-natural settings [25]. Importantly, our experimental design differed from prior studies [19,26] in that females were only briefly exposed to males each day under controlled settings, where we visually confirmed the occurrence of each insemination. This approach allowed us to minimize sexual harassment received by females. Sexual harassment involves relentless male pursuit of females, mountings and sometimes coercive matings, which reduce female fitness [27–30]. Given the potential energetic costs of wound healing and risks of infection associated with traumatic insemination in bed bugs [19,23], along with evidence from three meta-analyses suggesting a negative association between mating rate and longevity [7,16,31], we predicted that females inseminated at higher rates would experience a reduction in lifespan. We also predicted that higher rates of traumatic insemination would be associated with decreased rates of egg production since mating has been shown to stimulate more rapid reproductive senescence in other species [32,33]. In combination, we predicted that these negative effects of high insemination rates on longevity coupled with a reduction in reproductive rates would result in overall decreased lifetime reproductive success in females inseminated at high versus low rates.

Second, we experimentally manipulated female insemination status in order to critically test the effect of female insemination recency on male mountings, female avoidance, male rejection of females and traumatic insemination rates in realistic social network settings. Taking a social-network approach, we assessed the effect of females’ insemination status by observing replicate groups of 12 female and 12 male bed bugs in a large, semi-naturalistic arena where half of the females were manipulated to be recently inseminated (just before the test), while the other half were distantly inseminated (2 days prior to the test). This approach blended elements of controlled laboratory and ecologically relevant field studies to provide insights into the interplay between male pursuit and female avoidance strategies in a dynamic group environment. While social network analyses have predominately been used to quantify social relationships [34–36], recent research has illustrated their utility for quantifying sexual interactions at the realistic level of social groups [37–41]. We predicted that distantly and recently inseminated females would be mounted at equal rates because previous observations suggested that male bed bugs indiscriminately mount all bed bug-sized objects [18,42]. We also predicted that, owing to the cost of high- insemination rates, recently inseminated females would be more likely to avoid mounting males and hence be inseminated at lower rates than distantly inseminated females. Nonetheless, we also examined how often males aborted mounts directed at recently versus distantly inseminated females to account for the possibility of male mate choice [43–46].

Finally, we measured female avoidance under controlled settings, where females experienced successive daily inseminations over 6 days. We predicted that, as females receive an increasing number of inseminations, they would become progressively more resistant to male pursuit and insemination attempts, leading to longer insemination latencies and shorter insemination durations.

## 2. Methods

### (a) Study population and maintenance

We used descendants of bed bugs (*C. lectularius*) collected from four sites in southern Ontario between October 2019 and January 2020. We maintained the colony in two large 54 × 40 × 40 cm plastic storage bins kept at 27 ± 0.5°C and 60% relative humidity with lights off at 8.00 and on at 16.00. This reversed lighting schedule allowed us to conduct our experiments during the dark phase, when bed bugs are active. Within the plastic bins, we housed bed bugs in 85 ml spice jars each containing several strips of folded filter paper to provide a rough surface for walking and oviposition. Each jar contained roughly 50–150 bed bugs of the same life stage. We fed the colony weekly under red light with defibrinated rabbit blood (Hemostat Laboratories, Dixon, CA) using a Hemotek membrane-feeding system (Discovery Workshops, Accrington, UK). In all experiments, we generated virgin bed bugs by individually isolating recently fed fifth-instar bed bugs and grouping them into same-sex groups once they emerged as adults.

### (b) The cost of traumatic inseminations

To quantify the cost of repeated traumatic inseminations, we compared the lifetime reproductive output of female bed bugs that were either inseminated daily or weekly. We selected one insemination per day as our high rate based on previously observed rates of traumatic insemination in bed bugs [19,25,47]. Most notably, we previously observed bed bugs in a complex, semi-naturalistic environment in which females had ample room and protective crevices to avoid excessive male pursuit and access to blood meals every other day [25]. In this setting, females had a high average insemination rate of 0.89 ± 0.06 (mean ± s.e.) per day. As for our low-insemination-rate treatment, female bed bugs have been shown to continuously lay fertile eggs for up to 10 weeks after a single insemination [24], and thus once per week reflects a relatively low but likely sufficient rate of insemination. We first randomly assigned 20 one-week-old, virgin adult females into each treatment. We housed each female in a 35 mm Petri dish arena lined with filter paper and containing a dark shelter tent folded from a 1 cm × 1 cm square of blue construction paper. We fed all females from both treatments 6 days before they were inseminated for the first time. The next day and every following week for the remainder of the experiment, we fed all females by briefly grouping them by treatment. We chose to feed weekly as bed bugs feed every 6–7 days when provided ad libitum access to blood [19]. Twice a week, we moved each female into a fresh arena and then counted the number of eggs present in each recently occupied arena. We kept the old arenas with eggs for an additional 8 days and then counted the number of first instar nymphs produced by each female. Counting of both eggs and offspring was conducted by observers blind to female treatment.

Every day for the high-insemination-rate females and once a week for the low-insemination-rate females, we conducted controlled insemination trials by placing a single male bed bug that had not mated for at least 48 h into each arena and continuously inspected arenas to confirm that insemination occurred. We removed males immediately after they dismounted females to prevent additional inseminations. If insemination did not occur within 10 min, we added a second male to each arena without removing the first male. We needed a second male for 6.8% of insemination trials and once an insemination began, we immediately removed the excess male to minimize additional interactions. On days where the low-insemination-rate females were not to be inseminated, we introduced a single male that had not mated for at least 48 h with its copulatory organ superglued against its body to each arena. We then continuously observed these trials to ensure each female was mounted and pursued by males for approximately 2 min and to ensure that insemination did not occur. Note that, while we equalized male harassment of females between the treatments, we also minimized the harassment to a few minutes per day because such harassment decreases female bed bug fitness [27]. This allowed us to isolate the cost of traumatic insemination. On two occasions, we accidentally removed and discarded the focal female during insemination trials, resulting in a total sample size of 19 per treatment.

We ran all of our analyses using R v. 4.1.1 [48]. To compare survivorship between the high- and low-insemination-rate females, we fitted a Cox proportional hazard model using the *coxph* function from the *survival* package with days survived as the dependent factor and treatment as the independent factor [49,50]. Since we ended the experiment on day 77, when the final high-insemination-rate female died, the remaining 13/19 females from the low treatment were right-censored in the survival analysis. We analysed differences in egg production rate of living females using a generalized linear mixed model (GLMM) with a negative binomial distribution where the response variable was the number of eggs each individual female laid during the week. The model included an offset with the log of number of days during the week where each female was alive to account for occasions where females died mid-week, and thus had fewer than 7 days to lay eggs. If females were dead for the entire week, egg production was entered as NA. We included female treatment and week as fixed factors and arena number as a random factor. We next compared offspring production between females from each treatment by fitting a linear mixed model (LMM) with treatment and week as fixed factors and arena number as a random factor. Here, we wished to capture differences in female fitness, which encompasses longevity, and thus entered offspring production as zero for females even after they had died. Lastly, we analysed differences in egg hatch rates using a GLMM with a binomial distribution and proportion of viable eggs per female per week represented using the *cbind*() function in R to combine hatched and unhatched eggs as the dependent variable. Once again, treatment and week were included as fixed factors and arena number as a random factor. For all models, we verified fits by inspecting plots of model residuals.

### (c) Effects of female insemination status on female avoidance and male rejection

Here, we experimentally manipulated female insemination status in order to quantify female and male behaviours that lead to the high-insemination rates observed under semi-natural settings. We observed five replicate groups of 12 male and 12 female bed bugs, where half of the females in each group were experimentally manipulated to be distantly inseminated and the other half recently inseminated. The distantly inseminated females were inseminated 2 days prior to the test while the recently inseminated females were inseminated within 30 min of the start of the test phase. We first generated focal male and female virgin bed bugs that were each given a unique ID using paint from Sharpie oil-based paint markers after brief anaesthetization with CO_2_. We marked the bed bugs one week after they emerged as adults and began the first round of inseminations 3 days after marking.

To conduct controlled insemination trials, we individually placed focal female bed bugs into 35 mm Petri dishes lined with filter paper. We then introduced a single non-focal virgin male bed bug into each Petri dish while an observer ensured that insemination only occurred once. We first allowed non-focal males to inseminate all the 12 focal females. On the following day, we fed both male and female focal individuals. One day later, 30 min before the observation phase of the experiment, we generated the recently inseminated focal females by allowing a new set of non-focal males to inseminate six randomly selected focal females. The recently inseminated females received a total of two inseminations compared with only a single insemination in the distantly inseminated females so that these treatments reflected our semi-natural settings in which females received about one insemination per day. We also allowed each of the 12 focal males to inseminate a single non-focal female to partially deplete their sperm and seminal fluid reserves as a means of promoting male choosiness during observations and to better reflect the natural conditions where males are unlikely to be virgin. A single insemination in male bed bugs depletes approximately 12% of their sperm and 19% of their seminal fluid volume [51]. For all insemination trials, when insemination did not occur within 10 min, we added another virgin non-focal bed bug of the opposite sex to the arena to ensure insemination.

**Figure 1.**
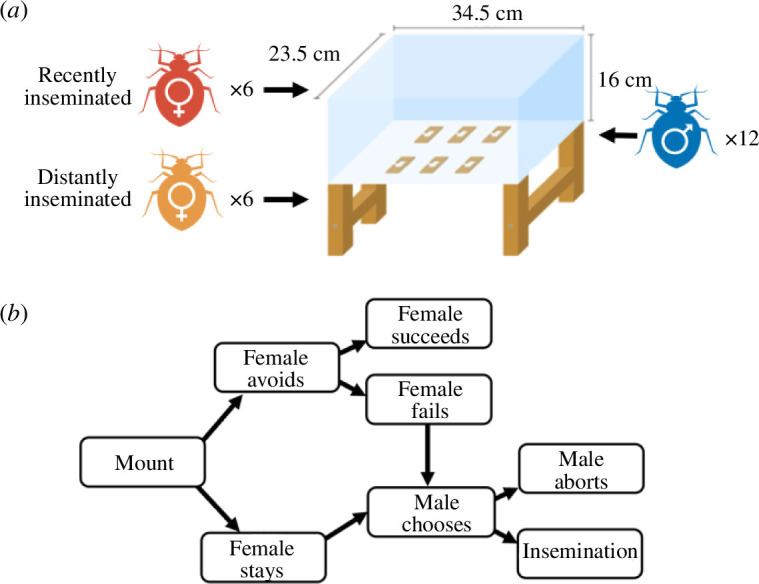
(*a*) Schematic overview of the experimental design and arena set-up including the positions of shelters. We observed interactions between six recently inseminated females, six distantly inseminated females and 12 standard males per replicate. (*b*) Flowchart illustrating how we scored sexual interactions and their outcomes.

Once all insemination trials were completed, we immediately placed the 12 mated males, 6 recently inseminated, and 6 distantly inseminated females into a 34.5 × 23.5 × 15 cm Plexiglas experimental arena lined with filter paper (figure 1*a*). In the arena, we placed six wooden shelters constructed from balsa wood slat segments covered with glass microscope slides (figure 1*a*). Each of these shelters are sufficiently spacious to accommodate all 24 adult bed bugs. We then documented all sexual interactions along with which individuals were involved in each interaction and the outcome of each interaction through continuous observation for 1 h while ensuring observers were blind to female treatment. The flowchart in figure 1*b* illustrates how sexual interactions and outcomes were scored. We recorded all mounts directed at females followed by whether the female attempted to avoid the mount. Attempted avoidance involved either running away or displaying the refusal postures described by Siva-Jothy [52]. If females did not avoid or failed to avoid a mount, we then recorded whether males aborted or proceeded with insemination. Inseminations were characterized by males remaining securely mounted with their abdomen curled underneath a female’s right abdomen for longer than 20 seconds [17]. Mounts, on the other hand, appear as a male quickly ‘jumping’ onto a female [19]. Both traumatic insemination and mounting are highly stereotyped and distinctive behaviours. Additionally, we validated our insemination criteria in a prior study where 92% of once-inseminated females produced offspring while no females in a non-inseminated reference group laid eggs [25].

We analysed female avoidance rate, male rejection rate and insemination rate as a function of female insemination status by constructing three GLMMs in R using the package *lme4* [53]. We verified model fits by visually inspecting plots of model residuals using the *DHARMa* package [54] and reported Wald *χ* ^2^ values derived from the *Anova* function from the *car* package [55]. We fitted binomial logistic regressions for all three models and included treatment as a fixed factor and replicate, male ID and female ID as random factors. Each of these three models had binary outcomes of mounts as the response variable. The female avoidance rate model assessed whether females attempted to avoid each mount directed at them. The male rejection rate model assessed whether males aborted each mount where they had the option to abort, meaning mounts successfully avoided by females were excluded. Finally, the insemination rate model assessed whether mounts resulted in insemination. We predicted that recent insemination would be associated with higher female avoidance rates, higher male rejection rates and, consequently, lower insemination rates.

We created all network visualizations with R v. 4.1.1 [48] using the *igraph* package [56]. For each of the five replicates, we created one mount and one insemination network. Mount networks display all mounts, including ones that eventually resulted in insemination. Each node represents an individual bed bug and node colours denote sex and treatment. In mount networks, weighted edges correspond to number of mounts that occurred between two individuals. In insemination networks, weighted edges correspond to the number of inseminations that occurred between two individuals. Directed edges indicate who initiated and who was the recipient of each sexual interaction. While males do mount other male bed bugs [42,57], we ignored these interactions during observations to ensure that we accurately captured all male–female interactions. Therefore, all edges in our sexual networks go from males to females. Lastly, node size corresponds to network strength (weighted degree), which is equivalent to the sum of all edge weights connected to a node. For example, larger female nodes in a given mount network represent females that received more mounts compared to smaller female nodes in the same network. We gave each individual within each replicate a unique letter ID and held node position constant between mount and insemination networks within the same replicate. These networks allow us to visualize which females were mounted and inseminated more based on overall differences in node size and connectedness (number of edges directed at each female). We did not test whether differences in network metrics were statistically significant as the analyses above already addressed our study questions and further analyses would have been redundant.

### (d) Effect of repeated traumatic inseminations on female avoidance

Here, we wished to test whether focal females show greater avoidance of males after receiving inseminations over 6 successive days. We video-recorded insemination trials for 13 focal females as they got inseminated once daily for six consecutive days. All females emerged as adults one week before the start of the experiment. We also ensured that all females were fed 1 day before the first day of the insemination trials. Each day, we placed each of the 13 females in a 35 mm Petri dish arena lined with filter paper and added a same-age male that had not mated for at least 48 h. Trials lasted until insemination ended or once 20 min had elapsed since the male was added. There were only two instances where focal females were not inseminated within the 20 min trial. For these instances, insemination latencies and duration were entered as NA while female avoidance rate was calculated as usual with trial duration lasting the full 20 min. One instance of a focal female not getting inseminated happened on the last day of recordings, so we did not need to ensure that the female was inseminated after the trial ended. In the other instance, we added a new male to the unmated female’s arena and ensured that insemination occurred so that we could continue using the female for the rest of the experiment. Between the daily insemination trials, we housed the focal females in an 85 ml jar containing folded strips of filter paper.

To video record trials, we used eight sixth-generation iPod Touches that captured two arenas at a time. Then, using BORIS observation software [58] to score the videos we recorded, an observer blind to both the treatment and day of each trial recorded the time of first encounter, the amount of time females spent either running away from males or in the refusal posture, and the start and end times of insemination, allowing us to calculate female avoidance rate, insemination latency and insemination duration. Every day, we also simultaneously recorded insemination trials for a new set of 13 virgin females that served as a reference group allowing us to control for day effects. These reference trials using virgin females allowed us to obtain baseline measures of insemination latencies, insemination durations and avoidance rates that account for day-to-day fluctuations in weather or environmental variables that have been shown to influence insect behaviour, even in controlled laboratory settings [59,60]. For each of our three response variables, we controlled for day effects by subtracting the daily mean of our virgin reference females’ scores from each focal female’s avoidance rate, insemination latency or insemination duration score. Because this subtraction occasionally resulted in negative values, we added a positive integer constant to each female score, allowing us to log-transform response variables to meet model assumptions. To calculate female avoidance rate, we looked at the proportion of time between first encounter and the start of insemination that a female spent either running away or in the refusal posture. This window of time captured the portion of the trial when males were pursuing females. Next, we fitted a LMM with the log of avoidance rate as the response variable, number of prior inseminations as a fixed effect and arena number as a random factor. Insemination latency was based on the time it took from first encounter to the start of insemination. We fitted a LMM with the log of insemination latency as the response variable, number of prior inseminations as a fixed effect and arena number as a random factor. Finally, we fitted a LMM with insemination duration as the response variable, number of prior inseminations as a fixed factor and arena number as a random factor. For all models, we verified fits by inspecting plots of model residuals.

## 3. Results

### (a) The cost of traumatic insemination

**Figure 2.**
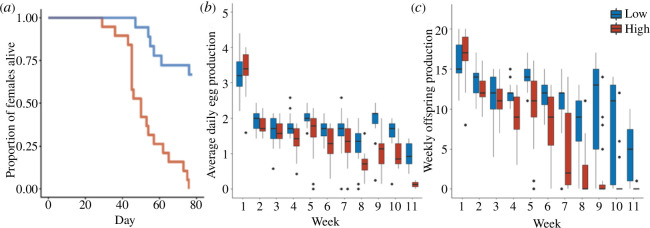
(*a*) Survival curves for females in the low, weekly insemination rate treatment group (blue; *n* = 19) and high, daily insemination rate treatment group (red; *n* = 19). (*b*) Average daily egg production rates for living females from the low- and high-insemination-rate groups. The initial sample sizes for each treatment are 19, but they gradually decrease with female death, culminating in 14 and 2 for the low- and high-insemination-rate groups, respectively. Bold horizontal lines indicate the medians, the boxes represent the IQR between the first and third quartiles, and the whiskers above and below each box represent values within ±1.5 of the IQR. Diamonds indicate outliers. (*c*) Weekly offspring production for females in the low- (blue) versus high- (red) insemination-rate groups. Note that this panel includes all females, both alive and dead (*n* = 19 females for each treatment).

The high-insemination-rate females had drastically lower survivorship than the low-insemination-rate females (Cox regression: Wald *χ* ^2^_1_ = 18.85, *p* < 0.0001; figure 2*a*). In fact, by the time all high-insemination-rate females had died, 68% of the low-insemination-rate females were still alive. Furthermore, the low-insemination-rate females produced eggs at a higher rate compared to high-insemination-rate females (GLMM: Wald *χ* ^2^_1_ = 28.03, *p* < 0.0001; figure 2*b*). Overall, the observed differences in longevity and egg production rate resulted in the low-insemination-rate females producing significantly more offspring than the high-insemination-rate females (LMM: Wald *χ* ^2^_1_ = 42.95, *p* < 0.0001; figure 2*c*). As we terminated the experiment before a majority of low-insemination-rate females had died, our results represent a lower estimate of the true cost of repeated traumatic inseminations. Finally, we found that hatch rate was similar in the low- than high-insemination-rate treatments (mean ± s.e. for proportion of eggs hatched: low = 0.98 ± 0.003, high = 0.97 ± 0.005; GLMM: Wald *χ* ^2^_1_ = 2.0, *p* = 0.16).

### (b) Effects of female insemination status on female avoidance and male rejection

**Figure 3.**
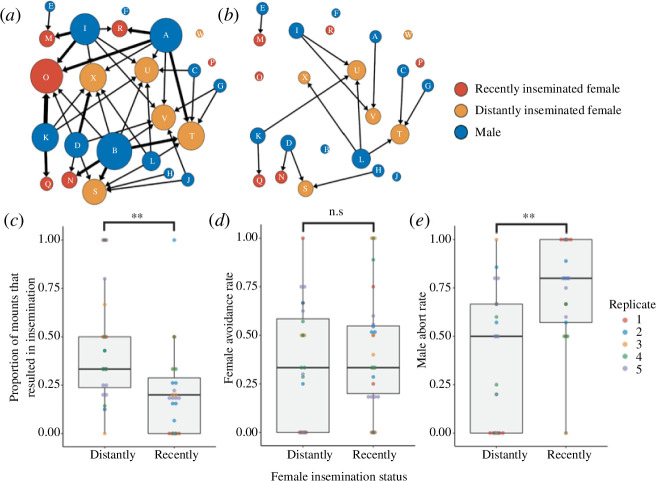
Weighted and directed sexual networks from a sample replicate depicting (*a*) mounts and (*b*) inseminations. Each node represents an individual bed bug and is labelled with a letter ID. The position of each individual is held constant in the two networks. Node colour depicts treatment and sex as indicated in the legend, while node size and edge width increase with strength (weighted degree). Sexual networks for all replicates are depicted in electronic supplementary material, figure S1. (*c*) Insemination rate, (*d*) avoidance rate and (*e*) male abort rate for distantly (*n* = 30) versus recently (*n* = 30) inseminated females. Each boxplot shows the raw values for each individual female and differently coloured data points refer to females from different replicates as indicated by the legend. Electronic supplementary material, figure S2 consists of boxplots depicting the total number of mounts and inseminations received by each female.

A greater proportion of mounts directed at distantly inseminated females resulted in inseminations (GLMM: Wald *χ* ^2^_1_ = 7.572, *p* < 0.01; figure 3*a–c*, electronic supplementary material, figures S1 and S2). This pattern was not driven by female avoidance behaviour as we did not detect any differences in the propensity to avoid mounts between females of the two treatments (GLMM: Wald *χ* ^2^_1_ = 0.99, *p* = 0.32; figure 3*d*). Males, however, were much more likely to abort insemination attempts with recently than with distantly inseminated females (GLMM: Wald *χ* ^2^_1_ = 6.43, *p* < 0.05; figure 3*e*).

### (c) Effect of repeated traumatic inseminations on female avoidance

**Figure 4.**
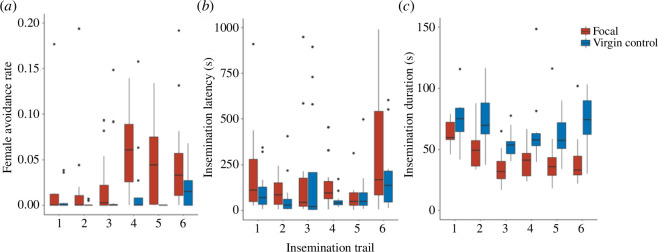
(*a*) Female avoidance rate, measured as the proportion of trial duration spent avoiding males, (*b*) insemination latency and (*c*) insemination duration for focal females that were inseminated once daily for six consecutive days (red; *n* = 13 females total, except for days 1 and 6 of the insemination latency and duration figures where *n* = 12, because one of the 13 females was not inseminated during the trial) and virgin reference females (blue; *n* = 13 females per day). The same group of 13 focal females were tested while a new group of age-matched virgin control females were used each day to control for day effects.

Female avoidance rates increased as a function of the number of prior inseminations (LMM: Wald *χ* ^2^_1_ = 5.35, *p* < 0.05; figure 4*a*). This was driven by a marked increase in avoidance behaviour following the females’ third consecutive insemination. However, increases in avoidance behaviour did not result in increased insemination latency over the course of the experiment (LMM: Wald *χ* ^2^_1_ = 1.12, *p* = 0.29; figure 4*b*). Lastly, as females received an increasing number of inseminations, the duration of each insemination decreased (LMM: Wald *χ* ^2^_1_ = 6.65, *p* < 0.01; figure 4*c*).

## 4. Discussion

Through tracking females that were inseminated at either daily rates or weekly rates, we found that daily rates of traumatic insemination resulted in a dramatic reduction in fitness. Females subjected to daily inseminations experienced reduced longevity, egg production and offspring production (figure 2). Next, we created replicate social networks of 12 male and 12 female bed bugs, where half of the females were recently inseminated while the other half were distantly inseminated. We found that a fewer proportion of mounts directed at recently inseminated females resulted in subsequent inseminations. However, to our surprise, recently inseminated females did not avoid males more frequently than distantly inseminated females. Instead, the observed difference in insemination rate was driven by males rejecting recently inseminated females at higher rates than they rejected distantly inseminated females (figure 3). To resolve the apparent contradiction between the high cost of multiple inseminations in our first experiment and lack of difference in female avoidance rates in the second experiment, we tracked female avoidance behaviour under controlled settings and found that females displayed more avoidance behaviour as they received more inseminations (figure 4). This increase in avoidance behaviour, however, did not result in longer insemination latencies (figure 4). Finally, insemination duration decreased over time (figure 4).

In the past few decades, the study of polyandry has received increasing scientific attention with an emphasis on the various potential fitness benefits and costs to females from mating with multiple males. The accumulation of studies on polyandry has demonstrated that a single mating typically does not maximize females’ fitness, with two meta-analyses reporting net fitness gains as high as 30–70% as a consequence of multiple mating [7,16]. In our current study, however, we found that high, daily rates of traumatic insemination dramatically reduced survivorship and lifetime offspring production (figure 2*a–c*). Since most of our low-insemination-rate females were still alive when we terminated the experiment, the true cost of daily insemination is probably higher than our result. Studies on multiple species of crickets, flies and beetles, however, have shown that polyandry can elevate female indirect fitness through increased egg hatch success rates [61–64]. Yet, our data also did not support indirect (genetic) benefits as we did not detect any differences in egg hatch rate between the low- and high-insemination-rate females. It remains possible that high-insemination-rate females benefitted from other indirect benefits that we did not quantify, such as increased offspring quality. However, such genetic benefits are unlikely to offset the >50% reduction in direct fitness we reported. Moreover, there is currently limited evidence that female multiple matings improve offspring performance metrics in other taxa [61]. Nonetheless, future studies should consider measuring offspring traits to examine potential indirect fitness benefits of high mating rates.

Overall, our results suggest that, while polyandry has generally been reported to be beneficial to females [7,16,31,65], some instances of polyandry may arise from sexual coercion and sexual conflict over mating rate, thus resulting in net fitness costs for females. For example, multiple mating has been shown to be costly in multiple species of beetles [66–70], fruit flies [32,33,71] and water striders [72]. It is currently unclear as to whether the variation in reported consequences of polyandry across studies reflects taxonomic differences or discrepancies in the mating rates and experimental conditions employed across studies. A key distinction between our study and most existing studies assessing the consequences of polyandry is that we exposed females to different mating rates throughout their lifetime until all females of one treatment died. Currently, estimates of the costs and benefits associated with multiple mating overwhelmingly come from experiments that compare only one versus two or one versus a small handful of matings, which likely do not capture realistic rates of female multiple mating in most species. In fact, only examining the small handful of studies that test higher rates of multiple mating reveals that higher rates of polyandry are often not beneficial [16] or even detrimental [7] to female fitness. This pattern has led researchers to suggest that females may experience an optimal intermediate mating rate, where further elevated rates of mating become deleterious [7]. Our results add to the small body of literature that test such elevated mating rates and demonstrate that high rates of polyandry can indeed be costly to females. To better understand the trade-offs associated with polyandry and critically test if females exhibit an optimal intermediate mating rate, future studies should examine female fitness under a broader range of mating rates that ideally capture what females in each species would naturally experience.

It is important to note that our results from Experiment 1 (figure 2) fall in line with Stutt & Siva-Jothy’s [19] but only partially with Morrow and Arnqvist’s [26] experiments on multiple traumatic inseminations in bed bugs. Both our study and that of Stutt & Siva-Jothy [19] found that high rates of insemination reduce female fitness while Morrow & Arnqvist [26] found a decrease in female survivorship but no overall differences in fitness for females inseminated at high versus low rates. This discrepancy can be attributed to the fact that we controlled for and minimized the effect of sexual harassment by exposing females to males for very short durations. Both prior studies continuously housed males with females, thereby exposing females to continuous harassment. Additionally, while both studies attempted to equalize harassment levels using males with glued genitals, we observed that males with glued genitals pursued females more relentlessly than control males (J.L.Y. & M.L.D., personal observation, 2022). Therefore, harassment levels were probably highly variable between studies and between treatments in the experiments by Stutt & Siva-Jothy [19] and Morrow & Arnqvist [26]. Sexual harassment has been shown to reduce elements of female fitness in a wide array of species [28,29,68,69,73,74]¸ including bed bugs [27]. Therefore, to minimize inconsistencies between studies, increase replicability and better isolate the effect of repeated matings, we suggest that future studies evaluating the fitness consequences of polyandry across taxa adopt protocols that both control for and reduce sexual harassment of females.

In our second experiment, which was conducted in a semi-naturalistic arena (figure 1*a*), we found that females’ insemination recency affected whether mounts directed at them resulted in inseminations (figure 3*c*, electronic supplementary material, figure S2). This pattern can be seen in the social networks (figure 3*a,b*, electronic supplementary material, figure S1), which depict similar node sizes and connectivity of females from both treatments in the mount networks, but larger and more connected nodes of distantly inseminated females in the insemination networks. While we predicted that the high cost of multiple inseminations would drive recently inseminated females to avoid mounts more frequently, we found no difference between the treatments (figure 3*d*). In this experiment, however, the recently inseminated females received only two inseminations prior to the test. We later found in our third experiment that females increased avoidance behaviour only following three successive daily inseminations (figure 4*a*). Hence, we likely required a higher number of successive inseminations in the second experiment to observe increased female avoidance rates. Alternatively, it is possible that running away is not an effective strategy. Nonetheless, given the immense amount of variation in females’ propensity to avoid mounts (figure 3*d*), it would be worthwhile to investigate other factors that may predict avoidance behaviour including female body condition and individual experience such as the outcomes of previous avoidance attempts. For instance, long-term exposure to males has been shown to increase swimming performance and aerobic capacity to facilitate escape from males in female Trinidadian guppies (*Poecilia reticulata*) [75].

Interestingly, we found that males aborted mounts more frequently with recently than distantly inseminated females (figure 3*e*). These results show that males respond to perceived sperm competition by foregoing insemination opportunities entirely, thus representing a clear example of male mate choice. Examples of male mate choice have been reported in an increasing number of species across insects, fishes, birds and mammals [43–45,76–78], and challenge the assumption from classical sexual selection theory that males should mate indiscriminately and at every given opportunity [6,79]. It is possible that the males in our study expected to encounter virgin or non-recently inseminated females in the near future and therefore conserved their limited sperm and seminal fluid reserves [51]. However, many additional factors like physiological limitations, sperm precedence patterns and variation in female quality can play a role in how males exhibit mate choice and respond to sperm competition. How these different factors interact remain poorly understood [80,81].

In our third experiment, we found that females increased the proportion of time spent avoiding males as they received six successive daily inseminations, with a notable increase on the fourth day, after females had already received three prior inseminations (figure 4*a*). First, this provides evidence that females possess plastic behavioural avoidance strategies based on their own sexual history, which may help mitigate the costs of repeated inseminations. Furthermore, our observation that females spent nearly no time avoiding males until the fourth daily insemination session suggests that up to three inseminations either increase or do not decrease their fitness. Across taxa, females may benefit from a small number of matings as opposed to one to protect against mating failure [82,83], increase the genetic diversity of their offspring [84–86] or enhance fecundity through receiving more beneficial ejaculate components [7,16,87]. Despite females spending more time avoiding males following their third consecutive insemination, insemination latency did not increase over successive daily trials (figure 4*b*). This was likely due to the small 35 mm arenas used in our experiment. Such limited space was necessary for quantifying subtle behaviours via close-up video recording, but limited females’ abilities to avoid males.

Lastly, we replicated previous findings showing that a female’s first insemination lasts significantly longer than subsequent inseminations (figure 4*c*) [88]. While we cannot rule out the possibility of females influencing males to terminate insemination, we have rarely observed changes in female behaviour that resulted in the terminations of inseminations. Therefore, seeing as insemination duration is positively correlated with the amount of ejaculate transferred [51], males appear to be investing more heavily in virgin females possibly because the absence of rival sperm signals lower sperm competition. This is consistent with our results from Experiment 2, where males displayed a preference for distantly over recently inseminated females (figure 3*e*), and our previous findings, where males preferred the social cues of virgin versus mated females [25]. However, currently, there is some limited evidence suggesting weak last-male sperm-precedence in bed bugs [19]. Yet, theoretical models predict greater investment in virgin females in mating systems with first rather than last-male sperm-precedence [80,81]. It is therefore evident that further research is required to uncover the patterns and mechanisms affecting sperm usage to understand the full scope of male and female sexual strategies under intense sperm competition.

In conclusion, we found strong evidence that high rates of one traumatic insemination per day in bed bugs result in dramatic fitness costs for females. Although females do increase their rate of avoiding sexual pursuit following multiple prior inseminations, why females endure insemination rates far above their apparent optimum remains unknown. Overall, these findings, coupled with our documentation of male mate choice, suggest that males predominantly control insemination rates in bed bugs. Our results also provide greater insight into how high rates of mating with multiple males can affect female fitness and male reproductive investment. Future work should focus on uncovering the direct and indirect fitness consequences of mating under a broader range of mating rates and examining the role that post-copulatory sexual selection mechanisms like sperm-precedence patterns play in male and female sexual strategies.

## Data Availability

Data available from Figshare [89]. Supplementary material is available online [90].
